# Assessment of Aseptic and Non-Aseptic Systems’ Influence on Basil (*Ocimum basilicum* L.) Microplants

**DOI:** 10.3390/plants13162313

**Published:** 2024-08-20

**Authors:** Oana Livadariu, Carmen Gabriela Constantin, Oana-Alina Boiu-Sicuia, Aurora Dobrin, Violeta Alexandra Ion

**Affiliations:** 1 Faculty of Biotechnologies, University of Agronomic Sciences and Veterinary Medicine of Bucharest, 59 Bd. Marasti, 011464 Bucharest, Romania; oana.livadariu@biotehnologii.usamv.ro; 2 Research Center for Studies of Food Quality and Agricultural Products, University of Agronomic Sciences and Veterinary Medicine of Bucharest, 59 Bd. Marasti, 011464 Bucharest, Romania; carmen.constantin@qlab.usamv.ro (C.G.C.); aurora.dobrin@qlab.usamv.ro (A.D.); violeta.ion@qlab.usamv.ro (V.A.I.)

**Keywords:** basil, microplants (microgreens/sprouts), aseptic/non-aseptic systems, substrate, biochemical and microbiological quality

## Abstract

Considering the current global climate and demographic conditions, combined with the growing demand for food diversification, the need for innovative functional foods that adhere to the principles of the circular economy is becoming clear. Therefore, this research aims to identify an appropriate cultivation system and growth substrate to maintain a high germination rate and produce basil aromatic microplants with superior quality traits that are entirely edible, together with the substrate. Microplants were grown in both aseptic (AS) and non-aseptic (NAS) systems. Both AS and NAS experiments were conducted in vitro using eco-innovative production technology. Moreover, various growth substrates were tested, such as perlite, agar, banana peel, peat, and their combinations. The analyses focused on the germination capacity, morphometric measurements, and biochemical analyses of the microplants. The results showed that the edible agar-based substrate, used in both AS and NAS, increased the germination capacity up to 95.00 ± 0.30%, while peat provided a germination capacity of only 12.07 ± 1.27% under AS conditions and 6.07 ± 0.35% under NAS conditions. Most biochemical analyses indicated that AS conditions are more suitable for basil microplant production, increasing the dry matter content, total phenolic content, total flavonoid content, and total antioxidant capacity compared to NAS conditions. These findings support the adoption of a new eco-innovative technology that provides organic basil microplants, which are fully usable along with the edible agar substrate.

## 1. Introduction

Designing, testing, and implementing technologies and systems to produce microplants that are free of microorganisms and rich in nutritional value and organoleptic flavor is becoming increasingly crucial in the modern socioeconomic context of Europe and around the world. This context is characterized by the accelerated depletion of natural resources, lifestyles adapted to both pandemic and post-pandemic realities, and a growing need for functional foods. Microgreens, with their fresh, delicate texture, distinct flavors, and high nutrient content, offer greater nutraceutical benefits than mature plants [1].

Basil (*Ocimum basilicum* L.) is one of the species sold either in the form of seeds for microgreen/sprout production or directly as microplants. The nutritional and functional properties of its seeds are associated with their flavor, taste, and aroma, which are highly valued by consumers [2,3]. As microgreens/sprouts (hereafter referred to as microplants), these attributes make basil a species with the potential to become a functional food with possible therapeutic implications. Research on microplant production is still in the early stages and is continuously evolving for both dicotyledonous and monocotyledonous species [4]. Furthermore, one of the emerging directions in this field highlights the need to optimize pre-harvest and post-harvest practices for the biofortification of microplants intended for the prevention or even treatment of chronic diseases [5].

Currently, microplants are produced and marketed locally and internationally. Although their production is generally successful, microplants are susceptible to microbial contamination, including phytopathogenic attack. Such infections can substantially reduce their quality or even compromise whole batches of products at any stage along the producer–retailer–consumer chain. According to the European Commission Directorate-General for Health and Food Safety in a Guidance document on the implementation of certain provisions of Regulation (EC) No. 852/2004 regarding the hygiene of foodstuffs, microplants are considered primary products of plant origin, along with grains, fruits, vegetables, and mushrooms. This document also outlines best practices for growing microplants, including their production, worker hygiene, and transport [6]. In light of these considerations, as well as the recommendations of the European Sprouted Seeds Association—ESSA [7], new technologies for producing basil microplants are needed.

Several technological aspects have been considered for improving microplant cultivation, including lighting [8], growth substrates [9,10], and production strategies [11]. Kwack et al. [12] tested various light intensities (0, 12.5, 25, 50, and 100 µmol m^−2^ s^−1^) of three monochromatic regimes (red, green, and blue) on six leguminous and brassicaceous microplants. Their findings confirmed that hypocotyl elongation was reduced and etiolation was prevented, particularly under blue light with increased intensity up to 100 µmol m^−2^ s^−1^. Similar effects were observed under red light, though predominantly in leguminous microplants. Fresh weight (FW) increased under red light at 100 µmol m^−2^ s^−1^; however, these conditions negatively affected the phenolic compounds of alfalfa and red radish sprouts [12]. Basil microplants grown under high-pressure sodium lamps, supplemented with monochromatic (blue and red) LED light, revealed higher accumulation of total phenolic compounds as well as total anthocyanins [13]. 

When studying the influence of growth media on microplant production, differences were observed in yield and quality parameters. Bulgari et al. [9] found higher accumulation of antioxidant compounds in red basil microplants when grown in vermiculite and jute fabric. However, coconut fiber decreased nitrate levels and improved sucrose content in red basil microplants compared to other growth substrates [9]. Increasing the substrate depth up to 6 cm positively influenced the harvestable FW of microplants and reduced the imbalanced moisture of the media [14]. Most growth parameters significantly influenced microplant yield and quality. Since microplants are promoted as being healthier than mature plants [11], their quality is highly important. The benefits of consuming microplants are due to their higher concentration of nutrients and antioxidants compared to mature leafy greens. To assess antioxidant properties, certain secondary metabolites should be considered, including polyphenols, flavonoids, vitamin C, and assimilatory pigments, all of which are highly relevant [15,16]. Mineral content is another important parameter indicating microplant quality and nutritional value [17]. Basil microplants are a rich source of minerals and provide larger amounts of calcium (Ca), potassium (K), and iron (Fe) compared to other microplants [18].

This study aims to present experimental findings related to the assessment of specific characteristics of aromatic basil microplants grown in both aseptic (AS) and non-aseptic systems (NAS) under LED lighting. Our approach integrates an eco-innovative cultivation system designed to reduce contamination risks by employing aseptic techniques, while also allowing for natural microplant development in non-aseptic conditions. We hypothesize that AS can effectively mitigate microbial contamination. Key parameters such as germination capacity, hypocotyl length, and leaf count were measured to evaluate microplant growth, as these factors directly influence economic outcomes [19]. Additionally, biochemical analyses were conducted to assess microplant quality based on factors such as total antioxidant capacity, total phenolic and flavonoid content, assimilatory pigments, and micro- and macronutrients. Furthermore, microbiological contaminations were examined to identify the potential phytosanitary issues associated with both aseptic and non-aseptic production methods.

## 2. Results

To introduce this novel concept, through experimental research, we examined the production technology within an eco-innovative cultivation system, which involves in vitro microplant production in an AS environment. This system involves the sterilization of seeds, substrates, and microplant growth containers, all conducted under HEPA-filtered airflow. The goal is to achieve a contaminant-free microplant production process, thereby mitigating microbial risks associated with manipulation or the microbial load present on materials such as containers, substrates, and seeds. In contrast, the NAS does not involve disinfection of seeds, lacks asepticization of substrates and containers, and grows microplants under ex vitro conditions. Both systems operate under similar LED-lit conditions, with variations in substrate composition, within the eco-innovative AS and the conventional NAS systems’ conditions [20,21,22].

### 2.1. Microplants Morphometry

#### 2.1.1. Germination Capacity

The experimental results for the germination capacity (GC), as outlined in Table 1, reveal that the highest average GC value (95.00 ± 0.30%) was recorded for the experimental variant consisting of the combination of AS with the substrate consisting of 100% agar (BV2). The lowest average GC value (6.07 ± 0.35%) was recorded for the experimental variant consisting of the NAS with a substrate of 100% conventional substrate (BV10). No germination was observed for substrates BV3, BV7, BV8, and BV9, likely due to the inhibitory effect of the banana-based substrate. Consequently, biochemical analyses were not conducted for four of the thirteen substrate variants (BV3, BV7, BV8, and BV9) in both AS and NAS due to the disruption caused by a lack of seed germination (in AS) or massive contamination with microorganisms (in NAS).

Among the experimental variants with only one component in their composition (BV1—100% perlite, BV2—100% agar, BV3—100% banana peel, and BV10—100% peat), BV2 in AS and BV1 in NAS achieved the highest basil GC at 95.00 ± 0.30% and 89.07 ± 0.72%, respectively. In contrast, the experimental variant BV10 resulted in low germination capacity (12.07 ± 1.27% in AS and 6.07 ± 0.35% in NAS), while BV3 produced no seed germination in both AS and NAS.

When comparing BV2 (100% agar) with BV4 (25% agar:75% perlite), BV5 (50% agar:50% perlite), and BV6 (75% agar:25% perlite) in terms of GC under both AS and NAS conditions, it is evident that a higher percentage of perlite leads to a lower GC. The germination percentage decreased by 13.30% with 75% perlite (BV6) in AS and by 42.14% in NAS, indicating that substrate sterilization plays an important role in cultivating microplants. Overall, BV2 consistently showed higher germination rates compared to BV4, BV5, and BV6 under both aseptic and non-aseptic conditions, highlighting its superior performance as a substrate for microplant cultivation.

When examining the utilization of a conventional substrate, represented by BV10 (100% peat), alongside BV11 (75% perlite:25% peat), BV12 (50% perlite:50% peat), and BV13 (25% perlite:75% peat), it becomes evident that higher peat content results in reduced germination capacity under both AS and NAS conditions. Comparing BV1 (100% perlite) with BV11 (75% perlite:25% peat), BV12 (50% perlite:50% peat), and BV13 (25% perlite:75% peat), the GC decreases gradually as the proportion of peat in the substrate increases. Specifically, BV11 shows a slight decrease compared to BV1, with further declines observed in BV12 and BV13, indicating a correlation between the peat content in the substrate and reduced GC. Although the seed supplier reported a high GC for the basil variety used, this trait may have been diminished or altered due to factors such as seed storage, the disinfection procedure used in the AS system, or the nature of the germination substrate.

#### 2.1.2. Hypocotyl Length

Hypocotyl length (HL) serves as a crucial indicator of microplant growth and development, particularly during the initial phases of germination and seedling establishment. As presented in Table 1, BV2 exhibited a longer HL (2.14 ± 0.59 cm) compared to BV1 (1.46 ± 0.27 cm) under AS conditions. Similarly, under NAS conditions, BV2 (2.98 ± 0.18 cm) displayed a longer HL compared to BV1 (2.33 ± 0.54 cm). BV4 showed a slightly longer HL than BV1 under both AS and NAS conditions; however, the differences were not significant.

Once again, among the experimental variants with only one component in their composition, the agar substrate demonstrated higher values compared to the other substrates. In AS, BV2 exhibited a longer HL (2.14 ± 0.59 cm), similar to BV4 (2.12 ± 0.46 cm), followed by BV6 (1.71 ± 0.29 cm) and BV5 (1.55 ± 0.08 cm). Likewise, in NAS, BV2 had the longer HL (2.98 ± 0.18 cm), similar to BV4 (2.60 ± 0.03 cm), followed by BV6 (1.97 ± 0.47 cm) and BV5 (2.43 ± 0.23 cm).

The differences in HL observed among peat substrates BV10, BV11, BV12, and BV13 under both AS and NAS conditions can be attributed to several factors, such as the cultivation system and substrate composition. BV11 exhibited the longest HL across both AS (4.46 ± 0.11 cm) and NAS (6.07 ± 0.10 cm), followed by BV12 in AS (4.54 ± 0.19 cm) and NAS (5.73 ± 0.29 cm). BV10 showed intermediate HL in both AS (3.04 ± 0.33 cm) and NAS (3.94 ± 1.43 cm). Conversely, BV13 displayed the shortest HL in both AS (3.46 ± 0.05 cm) and NAS (3.75 ± 0.72 cm). Significant differences were observed between BV11 and VB12 in NAS compared to the rest of the peat containing substrates. However, between BV10 and BV13 in NAS and BV11, BV12 and BV13 in AS, the differences are not statistically significant. 

A longer hypocotyl often suggests that the seedling has more energy reserves and is better able to push through soil or growth media to reach the surface. This can be advantageous for seedlings in competitive environments where they need to quickly establish themselves and compete for light and resources.

#### 2.1.3. Number of Leaves

The results presented in Table 1 regarding the number of leaves (NL) indicate that the highest average count (4.37 ± 0.17) was recorded for BV13, and the lowest average count (3.03 ± 0.10) was recorded for BV1 in the NAS cultivation conditions, regardless of the substrate composition (BV13 with 25% perlite and 75% conventional substrate or BV1 with 100% perlite). However, irrespective of the cultivation system, the maximum NL was observed with the same substrate, 25% perlite and 75% conventional substrate (BV13), in both AS (4.31 ± 0.11) and NAS (4.37 ± 0.17).

Comparing BV1, BV2, BV4, BV5, and BV6 for leaf count under AS and NAS conditions, BV6 consistently displayed the highest NL, followed by BV5, BV4, BV2, and BV1. Specifically, BV6 had a higher NL under both AS (3.84 ± 0.05) and NAS (3.86 ± 0.05) conditions, followed by BV5 (3.64 ± 0.05 in AS, 3.91 ± 0.02 in NAS), BV4 (3.50 ± 0.07 in AS, 3.70 ± 0.02 in NAS), BV2 (3.36 ± 0.04 in AS, 3.21 ± 0.08 in NAS), and BV1 (3.17 ± 0.18 in AS, 3.03 ± 0.10 in NAS). BV7 was not included in the comparison due to missing data.

Comparing BV10, BV11, BV12, and BV13 for NL, BV13 consistently showed the highest leaf count, followed by BV12, BV11, and BV10. Specifically, BV13 had the highest leaf count under both AS and NAS conditions, with no significant differences compared to BV12 in AS and NAS, and BV11 in AS. Overall, there was an increasing trend in the NL from BV1 to BV13 under both AS and NAS conditions, with BV13 consistently having the highest count. Additionally, the NL tends to be higher under NAS conditions compared to AS conditions for most variants.

### 2.2. Biochemical Evaluation of Basil Microplants

#### 2.2.1. Dry Matter Content

The accumulation of dry matter content (DM) represents an important physiological process that is related to crop yield and the average density of leaf tissues, and can also be related to the concentration of water-soluble carbohydrates [23,24]. The experimental results for DM content reveal that basil microplants grown in AS conditions with a substrate of 25% perlite with 75% agar achieved the highest accumulation, while the lowest was recorded in NAS conditions using a perlite substrate (Table 2). 

However, for the agar-based substrates, BV4 showed a notable increase in DM percentage under NAS conditions compared to AS conditions, while BV6 displayed higher DM percentages under AS conditions compared to NAS conditions. With the conventional substrates BV10, BV11, BV12, and BV13, there were no statistically significant differences in DM accumulation between NAS conditions and BV1, BV4, and BV5 in AS conditions. The substrate composition appears to have a significant impact on dry matter content compared to the choice between AS and NAS conditions. This is evident from the notable variations in dry matter percentages observed across different substrates, irrespective of whether the microplants were cultivated in AS or NAS conditions. Additionally, microplants grown on BV1 and BV11 in NAS conditions showed the same accumulation in DM as the microplants cultivated on BV1 and BV4 in AS conditions.

#### 2.2.2. The Assimilatory Pigments

The chlorophyll *a* content varied between 7.79 ± 0.23 mg/g in BV11 and 10.48 ± 0.03 mg/g in BV10 for NAS conditions (Table 2). In AS conditions, the assimilatory pigment accumulations varied between 7.91 ± 0.12 mg/g in BV5 and 12.22 ± 0.55 mg/g in BV6. Under AS conditions, microplants grown in a substrate of 25% perlite and 75% agar exhibited the highest chlorophyll *a* concentration in BV6, followed by BV4 and BV1. Conversely, under NAS conditions, basil grown on 100% peat had the highest chlorophyll *a* content, followed by BV1 and BV2. These findings highlight the variability in chlorophyll *a* production across different variants and growth conditions, suggesting the influence of substrate composition and cultivation environment on pigment synthesis. No statistically significant differences were found between chlorophyll *a* accumulation in BV5, BV11, BV12, and BV13 in NAS conditions and BV5 in AS conditions. 

The chlorophyll *b* content in microplants follows the same pattern as chlorophyll *a*; the microplants with the highest pigment accumulation were found in AS conditions in BV6, while the lowest accumulation was observed in NAS conditions in BV11. Among the substrates, BV6 consistently exhibited the highest levels under both AS and NAS conditions. Specifically, under AS conditions, BV6 had the highest chlorophyll *b* content, followed by BV4. Under NAS conditions, BV10 showed the highest chlorophyll *b* concentration, followed by BV1. These findings suggest that substrate composition significantly influences chlorophyll *b* production, with the BV6 substrate consistently promoting higher levels compared to others. 

The total chlorophyll content in microplants ranged from 13.74 ± 0.52 mg/g in BV11 substrate under NAS conditions to 22.70 ± 1.17 mg/g in BV6 substrate under AS conditions. The chlorophyll *a*/chlorophyll *b* ratio ranged from 1.14 for BV7 under AS conditions to 1.36 in BV12 under NAS conditions. This ratio between chlorophyll *a* and chlorophyll *b* provides insights into photosynthetic efficiency and nitrogen availability. A higher chlorophyll *a*/chlorophyll *b* ratio typically indicates plants adapted to environments with high light intensity, such as full sunlight, but with lower nitrogen availability [25].

#### 2.2.3. The Total Phenolic Content

When comparing the total phenolic content (TPC) of BV2, BV4, BV5, and BV6 under both AS and NAS conditions, as shown in Table 2, the following observations were made: under AS conditions, BV4 exhibits the highest TPC of 1.67 ± 0.16 mg GAE/g, followed by BV6 (1.56 ± 0.18 mg GAE/g), BV5 (1.49 ± 0.21 mg GAE/g), and BV2 (1.45 ± 0.08 mg GAE/g); Under NAS conditions, BV4 still maintains the highest TPC at 1.11 ± 0.14 mg GAE/g, followed by BV6 (1.22 ± 0.04 mg GAE/g), BV5 (1.19 ± 0.06 mg GAE/g), and BV2 (1.46 ± 0.06 mg GAE/g).

Among BV10, BV11, BV12, and BV13, BV12 exhibits the highest TPC under NAS conditions, with a value of 1.24 ± 0.32 mg GAE/g. Conversely, BV10 shows the highest TPC under AS conditions, with a value of 0.73 ± 0.05 mg GAE/g. BV11 and BV13 have lower TPC values compared to BV10 and BV12 under both AS and NAS conditions, with BV11 having a TPC of 0.58 ± 0.04 mg GAE/g and BV13 having a TPC of 0.41 ± 0.06 mg GAE/g.

From the comparison, it is evident that BV7 has the highest TPC under AS conditions (1.86 ± 0.08 mg GAE/g), while BV4 has the highest TPC under NAS conditions (1.67 ± 0.16 mg GAE/g). Conversely, BV10 has the lowest TPC under AS conditions (0.73 ± 0.05 mg GAE/g), and BV13 has the lowest TPC under NAS conditions (1.07 ± 0.18 mg GAE/g).

The TPC content varied among the microplants cultivated in AS conditions, ranging from 0.41 ± 0.06 mg GAE/g in BV13 to 1.86 ± 0.08 mg GAE/g in BV7. Similarly, in the NAS case, the microplants accumulated polyphenols ranging from 0.73 ± 0.05 mg GAE/g in BV10 to 1.46 ± 0.06 mg GAE/g in BV2. Furthermore, the microplants grown on BV1, BV2, BV4, BV5, and BV6 substrates under AS conditions showed similar polyphenol content to those grown on BV10, BV11, BV12, and BV13 under NAS conditions. 

#### 2.2.4. Total Antioxidant Capacity

Under AS conditions, BV2 demonstrates the highest total antioxidant capacity (TAC), measuring 18.99 ± 1.41 mg TE/g, while BV13 exhibits the lowest activity, recorded at 11.92 ± 1.30 mg TE/g. Conversely, under NAS conditions, BV6 displays the highest TAC at 26.00 ± 1.79 mg TE/g, whereas BV13 consistently demonstrates the lowest activity among all variants, registering 4.24 ± 0.70 mg TE/g. A noticeable positive correlation was observed between BV2 (21.77 ± 1.85 mg TE/g) and BV10 (22.23 ± 1.16 mg TE/g), with both displaying relatively higher values compared to BV1 (15.66 ± 0.72 mg TE/g).

When comparing classic substrates under NAS conditions, BV10 exhibits the highest TAC at 22.23 ± 1.16 mg TE/g, followed by BV12 (12.20 ± 0.47 mg TE/g), BV11 (6.44 ± 0.24 mg TE/g), and BV13 (4.24 ± 0.70 mg TE/g) successively, indicating variations in their antioxidant potential. 

Under AS conditions, BV2 demonstrates a moderate TAC of 18.99 ± 1.41 mg TE/g, while BV4 and BV6 exhibit a slightly lower TAC, with values of 14.98 ± 2.78 mg TE/g and 15.69 ± 0.66 mg TE/g, respectively. BV5 shows slightly higher activity than BV2, measured at 17.18 ± 1.59 mg TE/g.

Under NAS conditions, BV5 and BV6 display the highest TAC among the samples, with values of 22.98 ± 1.38 mg TE/g and 26.00 ± 1.79 mg TE/g, respectively. BV2 and BV4 also exhibit increased TAC compared to their respective values under AS conditions, measured at 21.77 ± 1.85 mg TE/g and 20.99 ± 1.98 mg TE/g, respectively.

Overall, BV6 consistently demonstrates the highest TAC, while BV13 consistently exhibits the lowest TAC across both AS and NAS conditions. No statistically significant differences were found between TAC in BV1, BV2, BV4, BV5 and BV10 in NAS conditions and BV2 in AS conditions.

#### 2.2.5. The Flavonoid Content

Under AS conditions, BV6 exhibited the highest total flavonoid content (TFC), registering 0.0038 mg RE/g, whereas BV4 displayed the lowest content at 0.0024 mg RE/10 g. Conversely, under NAS conditions, BV6 still demonstrated the highest TFC, while BV1 exhibited the lowest content (Table 2).

Comparing the TFC among BV2, BV4, BV5, and BV6 under both AS and NAS conditions revealed distinct trends. Specifically, under AS conditions, BV6 showcased the highest content at 0.0038 mg RE/g, followed by BV1 and BV 2 with 0.0034 mg RE/g, BV5 (0.0033 mg RE/g), and BV4 (0.0024 mg RE/g). 

Under NAS conditions, BV6 follows the same trend as in AS conditions, with the highest accumulation of 0.0044 mg RE/g. BV4 and BV2 had the same content of 0.0042 mg RE/g, followed by BV5 at 0.0038 mg RE/g and BV1 at 0.0033 mg RE/g. Microplants from BV1, BV2, and BV5 in AS condition showed the same accumulation in TFC as BV10 under NAS conditions. Basil grown on BV1, BV2, and BV5 substrates exhibited similar flavonoid content both in AS and NAS conditions.

#### 2.2.6. Macro- and Microelements Content in Microplants

The results of the elemental analysis of basil are presented in Table 3. Among the macroelements found were sodium (Na), magnesium (Mg), phosphorous (P), K, and Ca, while microelements included manganese (Mn), copper (Cu), and zinc (Zn). Under NAS conditions, the Na content varied between 45.21 ± 0.50 mg/kg (BV11) and 561.60 ± 16.95 mg/kg (BV13), while under AS conditions, it ranged from 716.83 ± 61.64 mg/kg (BV2) to 1296.51 ± 24.67 mg/kg (BV5). Significantly different Na content was observed in microplants obtained through cultivation on BV1, BV2 (AS), and BV10 cultivation substrates. The higher amounts of Na under AS conditions are likely due to the sterilization method used for the substrate. Similarly, Mg content varied between 257.73 ± 38.71 mg/kg (BV6) and 606.67 ± 29.64 mg/kg (BV11) under NAS conditions, while under AS conditions, it ranged from 219.30 ± 8.28 mg/kg (BV5) to 288.94 ± 10.50 mg/kg (BV4). No statistically significant differences were highlighted between BV1, BV2, BV4, and BV6 under AS conditions and BV2, BV4, BV5, BV6, and BV10 under NAS conditions. 

Regarding P content, values ranged from 537.46 ± 32.21 mg/kg (BV10 to BV12) to 845.24 ± 28.16 mg/kg (BV4), while under AS conditions, the content ranged from 689.79 ± 38.06 mg/kg (BV2) to 810.98 ± 30.80 mg/kg (BV6). No significant differences were observed between BV1, BV2, BV11, and BV 13 under NAS conditions and BV1, BV2, and BV5 under AS conditions.

K content varied between 1426.63 ± 60.17 mg/kg (BV2) and 3816.39 ± 223.86 mg/kg (BV13) under NAS conditions and between 995.65 ± 66.41 mg/kg (BV1) and 1467.40 ± 75.47 mg/kg under AS conditions. Statistically, the results were no significantly different for BV2, BV4, BV5, and BV6 (AS) and BV1, BV2, BV5, and BV6 (NAS). Significantly different K content was observed in microplants obtained through cultivation on BV12 and BV13.

Ca content varied between 253.40 ± 25.22 mg/kg (BV1) to 1872.45 ± 92.64 mg/kg (BV11) under NAS conditions, while under AS conditions, it ranged from 131.85 ± 3.02 mg/kg (BV5) to 228.54 ± 54.86 mg/kg. Statistically, there were no significant differences among BV1-BV6 under both AS and NAS conditions. BV11-BV13 under NAS conditions also showed no significant differences. However, significant differences in Ca content were observed in microplants cultivated on BV10.

Mn content ranged from 2.64 ± 0.17 mg/kg (BV6) to 44.42 ± 0.58 mg/kg (BV13) under NAS conditions, while under AS conditions, it varied from 2.16 ± 0.09 mg/kg (BV2) to 3.10 ± 0.05 mg/kg (BV5). According to Table 3, statistically similar results were observed between BV1-BV2 and BV4-BV5 under AS conditions. No significant differences were found for the BV6 variant. Significant differences in Mn content were observed in microplants cultivated on BV13.

Cu content ranged from 1.53 ± 0.03 mg/kg (BV5) to 2.80 ± 0.17 mg/kg (BV13) under NAS conditions and from 2.16 ± 0.09 mg/kg (BV2) to 3.10 ± 0.05 mg/kg (BV5) under AS conditions. Similar results were identified for BV1, BV5, and BV6 variants grown under NAS conditions. No significant differences were recorded between AS and NAS conditions for BV1-BV10, and BV 12 regarding Cu content. 

Finally, Zn content varied between 8.88 ± 0.82 mg/kg (BV10) and 16.01 ± 0.18 mg/kg (BV4) under NAS conditions and from 7.06 ± 0.51 mg/kg (BV5) to 8.83 ± 0.52 mg/kg (BV4) under AS conditions. No significant differences were found between BV1, BV2, BV4, BV5, and BV6 under AS conditions and BV10 under NAS conditions. Zn content fell below the limit of detection (LOD) of the method for BV11, BV12, and BV13. Significant differences in Zn content were observed in microplants cultivated on BV4 (NAS).

### 2.3. Microbial Contaminants of Cultivation Technologies

Basil microplants grown in the NAS were highly susceptible to microbial contamination. However, the disinfection procedures applied to the seeds, substrate, and jars, along with the phytosanitary hygiene measures in the AS, effectively reduced microbial contamination in the microplants compared to the NAS (Table 4). As a result, only a few accidental contaminations occurred in the AS.

In the AS, only a few microbial infections were encountered. *Acremonium* and *Penicillium* molds, which are opportunistic airborne contaminants, were likely introduced during sowing. The other fungal contaminants, *Curvularia* sp. and *Fusarium* sp., are phytopathogenic species that might have originated from asymptomatic infected seeds, which were only surface disinfected. However, it cannot be ruled out that some *Fusarium* chlamydospores remained viable due to incomplete disinfection of the plant growth substrates. In the non-aseptic production system, *Phoma* sp. infections were more common among the experimental variants, while *Fusarium* sp. was present only in substrates containing peat.

## 3. Discussion

Since the specifics of seed germination did not allow for the synchronous germination of all seeds, and considering that this experimental research aims to scientifically demonstrate the feasibility of a new concept with potential technological application for basil microplant production, the morphometric measurements included all obtained microplants. This approach reflects a method similar to that of final consumers, who consume microplants at various stages of development, including microgreens and sprouts.

Basil GC was significantly influenced by the seeding substrate and less so by the cultivation system. Among the tested substrates, perlite and edible agar prove to be more effective for ensuring high germination. Other studies have also investigated the impact of substrate on basil germination. Cicek et al. [26] compared a peat/perlite mixture to various peat/perlite combinations supplemented with organic manure at concentrations of 2%, 4%, and 6%. They found that the most suitable substrate for basil was a peat/perlite mixture supplemented with 6% bat guano [26]. 

Biofortified substrates have been considered for microplants [27,28,29], but they primarily influenced the nutritional value of microplants rather than the GC of the seeds. In addition to traditional substrates, fiber-based alternatives such as coconut fiber, jute fabric, and cellulose sponge have been tested [9]. Hydroponics and aeroponics have also been explored as alternatives to traditional germination substrates for microplants [30,31,32], particularly in vertical agriculture. However, these methods are more susceptible to microbial safety issues [33]. 

Currently, hydrogel-based substrates are gaining attention for microplant cultivation [32]. Our study also considered this approach by using edible agar. Among these biopolymer matrixes are agar-based gels, phytagels, sodium alginate-based gels, and derivates of cellulose and chitosan. Biopolymer hydrogels are suitable for urban agriculture as they mitigate food safety risks, can prevent plant pathogen contamination, maintain appropriate humidity to reduce drought stress, allow for biofortification, and, in some cases, are even edible. Importantly, they do not affect germination [32]. Besides the growth substrate, basil seed germination can also fluctuate depending on the variety [34].

Basil HL can be influenced by seeding density [35] and lighting conditions [36], including light intensity [37], light spectrum [38], and photoperiod [39]. Green and purple basil microplants, grown in similar lighting as in this study, had HLs of 3.76 ± 0.05 cm and 4.52 ± 0.06 cm, respectively [40]. However, under iodine biofortification conditions, the HL slightly increased for green basil but decreased for purple basil. These findings suggest that not only do growth conditions affect microplants’ HL, but the plant variety also plays a role. Compared to other species, basil microplants generally exhibit slower growth rates [36]. For instance, lemon basil and red sweet basil varieties developed their cotyledonal and first true leaves much more slowly [36,41].

The experimental results for the DM content, shown in Table 2, indicate the highest average value of 6.89% ± 0.29% for basil microplants grown under AS conditions using BV6 substrate. The lowest DM content (4.70 ± 0.28%) was recorded under NAS conditions using the BV1 substrate. These results align with Ghoora et al. [42], who reported DM content for microplants ranging between 5.26% and 10.60%, with 5.72% for the French basil variety. Kyriacou et al. [43] found DM values between 3.89% and 6.05%, with 4.74% for purple basil and 5.26% for green basil. In addition to the highest DM content, basil grown on BV6 also exhibited the highest levels of Chl *a*, Chl *b*, total chlorophyll, and TPC. Hallmann et al. [44] observed that the cultivation substrate affects assimilation and photosynthesis rates, which can lead to higher DM content in plants grown on organic substrates compared to conventional ones. In our case, adding 75% organic agar to perlite yielded the best results. According to Fayezizadeh et al. [45], there is also a correlation between chlorophyll, TPC, and TAC. They noted that light absorbed by the assimilatory pigments can influence cell metabolism and the synthesis of polyphenols and antioxidant compounds. For chlorophyll *a*, we observed lower values ranging from 7.79 mg/g to 10.57 mg/g, compared to Othman et al. [46], who reported values from 30 mg/g to 35.38 mg/g, with 31.66 mg/g for red basil and 33.7 mg/g for green basil microplants. For chlorophyll *b*, however, our values ranged from 5.94 mg/g to 10.48 mg/g, which are higher than the 4.45 mg/g to 6.01 mg/g reported by Othman et al. [46]. The chlorophyll content ratio of basil microplants was consistent with the findings of Fayezizadeh et al. [45], who reported values between 0.35 and 1.18. Similarly, Ghoora et al., 2020 [42] observed ratios between 1.30 and 2.15. An increase in Chl *a*/*b* ratio can be correlated with a response to higher intracellular light intensity [25].

For total flavonoid content, the highest average values were recorded as 0.0037 RE mg/g and 0.0044 mg RE/g in experimental variants that combined 25% perlite and 75% agar, under both NAS and AS conditions. These values are higher compared to those from the BV1 and BV2 cultivation systems (Table 2). These findings support the statement by Fayezizadeh et al. [45], who suggest that flavonoid biosynthesis is regulated by nitrogen availability through the allocation of photosynthetic carbon linked by the shikimate pathway, and that flavonoids are directly related to phenolic compounds and the antioxidant capacity of basil microplants.

The TPC in microplants cultivated on NAS substrates ranged from 0.41 mg GAE/g on BV13 to 1.46 mg GAE/g on BV2. For those grown on AS substrates, polyphenol accumulation varied from 1.34 mg GAE/g on BV1 to 1.67 mg GAE/g on BV4. These results align with those of Fayezizadeh et al. [45], who reported TPC between 135.88 and 1463.79 mg GAE/100 g across 21 basil microplant cultivars and genotypes. Variations in polyphenol content are influenced by the substrate, cultivation conditions, and genetic factors.

Both low and high photosynthetic photon flux densities (PPFs) can negatively impact growth and antioxidant accumulation. A PPF of 110 μmol m^−2^ s^−1^ impairs normal growth and reduces the nutritional value of microplants, while a PPF of 545 μmol m^−2^ s^−1^ can induce mild photostress [11]. Additionally, research has shown that purple leaf basil is more sensitive to UVA exposure from LEDs compared to green leaf basil, leading to decreased antioxidant properties [38]. While light type and intensity are primary factors influencing the total antioxidant capacity of microplants, substrate composition also affects the concentration of bioactive components and antioxidant activity. Comparing these findings with previous research reveals that basil microplants exhibit a range of antioxidant capacities comparable to those found in various plant genera. While fully characterizing basil seed and microplant extracts is challenging, it is evident that their antioxidant capacity is primarily attributed to phenolic compounds, along with other secondary metabolites such as carotenoids and volatile oils [2].

According to Bhaswant et al. [47], basil microplants are emerging as a valuable food source, and their mineral composition, including micronutrients like Fe, Zn, K, Ca, N, P, S, Mn, Se, Mo, and others is still under exploration. In our case study, where we screened 23 macro- and microelements, the main macronutrients in the microplants were Na, Mg, P, K, and Ca, while micronutrients included Mn, Cu, and Zn. Other elements, such as Fe, Se, Mo, Co, and so on, were below the limit of detection of our method (<LOD). 

The choice of growth substrate significantly impacts the yield and quality of microplants and is a key factor in the production process [9]. For example, the application of ZnO has been shown to influence chlorophyll and phenolic content [48]. Additionally, light intensity may also play a significant role [49]. Di Gioia et al. [17] found that the content of K in basil microplants ranged between 296.41 ± 20.5 mg/100 g and 347.79 ± 75.3 mg/100 g, which is comparable to our results in BV11 (2961.41 ± 192.20 mg/kg, NAS) and BV13 (3816.39 ± 223.86 mg/kg, NAS). They also reported Ca content of 141.67 ± 32.0 mg/100 g, similar to our findings in BV1 (141.98 ± 0.43 mg/kg, AS). Our P content results aligned with their range of 56 to 57 mg/100 g. The Mn content in BV1-BV6 was comparable to 0.24 g/kg DW reported by Bulgari et al. [41] for basil grown in an aquaculture system, though BV10-BV13 exhibits quantities ten times higher. Trace elements such as Cu, Zn, and Se play important roles as components or cofactors of antioxidant enzymes. According to Zhang et al. [5], Zn content in microplants ranges from 4.76 mg/kg to 29.12 mg/kg. Our results align with this range, showing Zn content between 7.06 mg/kg and 12.57 mg/kg.

Several phytopathogens have been noted as occurring in plants [50]. *Curvularia lunata* along with other related phytopathogenic species is known to be transmitted through seeds [51,52]. *Fusarium* sp. can also be present asymptomatically in seeds, without causing phytosanitary problems in subsequent crop cycles [53]. Moreover, studies on different disinfection methods for plant growth substrates have shown that hot air sterilization via injection is more effective in controlling *Fusarium*-resistant spores compared to pan steam treatment. This method can reduce chlamydospore viability to 40–80% of the initial load [54].

## 4. Materials and Methods

### 4.1. Biological Material and Sterilization

The biological material used consisted of green basil ‘Zena’ variety seeds (organic and untreated), obtained from a commercial source, Italian Sprout Ltd. (47521, Cesena (FC), Italy) [21]. This variety was chosen because it is commercially approved in the European Union for microplant production and is organic certified. The GC of basil in NAS is generally 60–80% [55], but for this variety, it is up to 90% according to the supplier [21]. High germinability seeds are preferred to ensure an optimal starting point for testing new microplant production technology in AS.

Germination and sprouting occurred under AS and NAS conditions. In the first case, the seeds were sterilized with a 10% sodium hypochlorite (Biovegan GmbH, Bonefeld, 56579, Germany) solution followed by five consecutive washes in distilled water to remove any chlorine residue from the seed surface [21]. This disinfection procedure ensures the removal of any potential contaminants present on the seed surface, which is an essential step in initiating aseptic production technology. In the case of the NAS, the seeds were not sterilized. In both situations, the seeds were germinated in glass containers (63 mm diameter, 300 mL capacity), which are transparent and recyclable [20,21,22]. 

### 4.2. Light Sources

White LEDs (Nichia, Tokushima 774-8601, Japan) were used as the light source [20,21,22,56,57] with a 16/8 h light/dark photoperiod. These white LEDs were preferred because they provide a continuous spectrum, which enhances the biomass production of basil microplants [56]. After 28 days, the microplants matured and underwent analysis [55]. The technical characteristics of the LEDs are as follows: a light flux of 1140 lm, a color temperature ranging from 2000 to 6500 K, tunable white light color, power consumption of 12.7 W, and a voltage of 220 V [20].

Throughout the experiment, the microplants were grown under artificial light with a measured PPFD (photosynthetic photon flux density) in the growth chamber of 281 ± 5.5 μmol m^−2^ s^−1^ and an air temperature of 21 ± 2 °C [21,22], a CO_2_ concentration of 350 ppm, and air relative humidity ranging from 50% to 70%.

### 4.3. Experimental Variants

In each of the two experimental cultivation systems (AS and NAS), in the presented research, we utilized 13 experimental substrate variants (Table 5). Each experimental variant was replicated three times. Perlite (Marcoser, Matca, 807185, Romania) was chosen due to its widespread use in horticultural applications. Perlite is nutritionally neutral and ensures good aeration and drainage of the substrate. Additionally, it can be easily removed from the roots during harvesting and can be recycled. Another selected substrate was edible agar (Biovegan GmbH, Bonefeld, 56579, Germany), a natural, organic agar used to stabilize the rooting support. This ensures that the microplants are fully consumed along with their rooting substrate. Organic banana peel (Port International Organics GmbH, Hamburg, 20097, Germany) is also classified as edible. Rich in K and P, banana peel can provide a nutritious growth substrate for the microplants. Additionally, this waste is recycled in a beneficial manner to support microplant production, contributing to the circular economy. Although peat is not categorized as an edible substrate, it was used as a comparative growth medium due to its widespread horticultural application as a conventional rooting support. The peat used was provided by BIOFLOR.BC SRL (Bacau, 600354, Romania). 

### 4.4. Morphometric Measurements of the Microplants

The morphometric measurements of the microplants included germination capacity (GC), hypocotyl length (HL), and number of leaves (NL). HL was measured per microplant using an EPSON Model Expression 11000XL scanner along with image analyzer software (WinFOLIA^TM^ Pro by Regent Instruments Inc., Quebec, QC, G2G 1B5, Canada). HL was selected for measurement due to its importance in assessing seedling emergence characteristics, especially when comparing various growth substrates [58]. Similarly, NL was recorded using the same scanner as it is a key growth parameter [59] that can reveal differences between the growth substrates. All morphometric determinations were performed on the final day of cultivation, regardless of the system used for microplant production.

$$GC(\%)=\frac{n_{g}}{n_{t}}\times 100$$

Abbreviations: *n_g_* = the number of germinated seeds and *n_t_* = the total number of seeds.

### 4.5. Plants’ Biochemical Evaluation

To evaluate the bioactive constituents of the fresh microplant biomass, the plants were removed from the rowth container, and the entire substrate was thoroughly washed off. The biochemical analyses conducted included assessments of dry matter (DM), assimilatory pigments (chlorophyll *a* (Chl *a*), chlorophyll *b* (Chl *b*), total chlorophyll (sum of Chl *a* and Chl *b*), and the Chl *a*/*b* ratio), total phenolic content (TPC), total flavonoid content (TFC), total antioxidant capacity (TAC), and macroelements (Na, Mg, P, K, and Ca) as well as microelements (Mn, Cu, and Zn).

For DM, the microplants were dried using a Memmert UN 110 oven (Memmert GmbH+Co. KG, Dresden, Germany) at 105 °C for 5 h [60], and the results were expressed as a percentage. For analysis of Chl *a*, Chl *b*, total Chl, and the Chl *a*/*b* ratio, the microplants were subjected to extraction with 80% acetone solution [61]. The extracts were analyzed using a Specord 210 Plus spectrophotometer (Analytik Jena GmbH+Co. KG, Jena, Germany) and expressed as mg/g.

To determine TFC, TPC, and TAC, the fresh microplant samples were extracted using a 70% methanol solution. TFC was characterized using the aluminum chloride method [62] and expressed as mg RE/g (rutin equivalents), TPC was assessed using the Folin–Ciocâlteu method [63] and expressed as mg GAE/g (gallic acid equivalents), and TAC was measured using the DPPH Radical Scavenging Assay [64] and expressed as mg TE/g (trolox equivalents). All three methods were performed using a Specord 210 Plus spectrophotometer (Analytik Jena GmbH+Co. KG, Jena, Germany).

For macro- and micronutrient analysis, the fresh samples were digested using a mixture of nitric acid and hydrogen peroxide in a 4:1 ratio. The samples were then analyzed using an Agilent ICP-MS 7700 (Agilent Technologies, Santa Clara, CA, USA) with MassHunter 4.3 Workstation software version C.01.03 (Agilent Technologies, Santa Clara, CA, USA) [65] and the results were expressed as mg/Kg.

### 4.6. Microbiological Analyses

The fungal contaminants on the microplants were microscopically examined, either on fresh slides or on Potato Dextrose Agar (PDA, Merck™, Darmstadt, Germany) in Van Tieghem cells. When grown on PDA, incubation was carried out at room temperature for up to 10 days. The specimens were examined under a light microscope (Nikon Eclipse E200LED MV R, Nikon Corporation, Tokyo, Japan) using 10× or 40× objectives. Some samples were stained with cotton blue to highlight their sporulation forms. Fungal identification was carried out based on their microscopic characteristics [66].

### 4.7. Statistical Procedures

The statistical analysis was conducted based on the following hypotheses (H) at a significance level of 0.05: (H1) biochemical parameters are influenced by the cultivation variant, (H2) the cultivation system affects the biochemical parameters, and (H3) there is interaction between the cultivation variant and the cultivation system. A two-way ANOVA was applied, with the biochemical characteristic as the dependent variable and the cultivation variant and system as fixed factors. The model included the main effects of the system and variants, as well as their interaction. Tukey’s post hoc test was used to evaluate pairwise differences, while Bonferroni correction was applied for estimating marginal means. Statistical analysis was performed using SPSS software (version 26.0), and the results are presented as the mean ± standard deviation (SD).

## 5. Conclusions

The experimental results obtained from comparative testing under aseptic (AS) and non-aseptic (NAS) conditions provide insights into basil microplant production and the impact of both intrinsic and extrinsic variables. Morphometric measurements, including germination capacity, hypocotyl length, and leaf count, highlight the need to experimentally correlate cultivation systems (AS or NAS) with different substrate types, such as perlite, agar, and banana peels, and conventional substrates (peat). This correlation is crucial for identifying the optimal conditions for basil microplant growth.

Biochemical analyses assessed dry matter, total antioxidant capacity, total phenolic and flavonoid content, assimilatory pigment content (chlorophyll *a*, chlorophyll *b*, total chlorophyll, and the chlorophyll *a*/chlorophyll *b* ratio), and macro- and micro-element content (Na, Mg, P, K, Ca, Mn, Cu, and Zn). The results based on the characterization methods revealed fluctuations in optimal growth conditions based on the cultivation system and substrate type, highlighting the importance of experimental correlation to achieve the desired biochemical quality.

Microbiological analyses indicated increased microbial contamination in NAS-grown basil microplants, suggesting that AS conditions are superior in preventing contamination-related losses. Integrating morphometric, biochemical, and microbiological data, AS cultivation with the BV2 substrate (100% agar) emerges as an effective technology for producing aromatic basil microplants. This approach not only supports their use as a functional food but also leverages the benefits of this edible substrate.

## Figures and Tables

**Table 1:** Morphometric measurements (mean ± standard deviation) of basil microplants in the cultivation substrate.

	AS			NAS		
Variants Cod	GC (%)	HL (cm)	NL	GC (%)	HL (cm)	NL
BV1	92.07 ± 0.72 ^b^	1.46 ± 0.27 ^e^	3.17 ± 0.18 ^gh^	89.07 ± 0.72 ^c^	2.33 ± 0.54 ^de^	3.03 ± 0.10 ^h^
BV2	95.00 ± 0.30 ^a^	2.14 ± 0.59 ^de^	3.36 ± 0.04 ^g^	88.53 ± 1.41 ^c^	2.98 ± 0.18 ^cd^	3.21 ± 0.08 ^h^
BV3	n.a.	n.a.	n.a.	n.a.	n.a.	n.a.
BV4	78.77 ± 3.45 ^ef^	2.12 ± 0.46 ^de^	3.50 ± 0.07 ^f^	46.93 ± 4.01 ^h^	2.60 ± 0.03 ^de^	3.70 ± 0.02 ^d^
BV5	81.90 ± 0.76 ^e^	1.55 ± 0.08 ^e^	3.64 ± 0.05 ^e^	56.30 ± 1.82 ^g^	2.43 ± 0.23 ^de^	3.91 ± 0.02 ^c^
BV6	84.00 ± 2.62 ^de^	1.71 ± 0.29 ^e^	3.84 ± 0.05 ^cd^	72.30 ± 2.46 ^f^	1.97 ± 0.47 ^e^	3.86 ± 0.05 ^c^
BV7	86.83 ± 1.42 ^cd^	1.93 ± 0.15 ^e^	3.05 ± 0.09 ^h^	n.a.	n.a.	n.a.
BV8	n.a.	n.a.	n.a.	n.a.	n.a.	n.a.
BV9	n.a.	n.a.	n.a.	n.a.	n.a.	n.a.
BV10	12.07 ± 1.27 ^j^	3.04 ± 0.33 ^d^	3.81 ± 0.09 ^cd^	6.07 ± 0.35 ^k^	3.94 ± 1.43 ^bc^	3.71 ± 0.08 ^d^
BV11	27.13 ± 1.60 ^i^	4.46 ± 0.11 ^c^	4.23 ± 0.14 ^a^	26.80 ± 4.65 ^i^	6.07 ± 0.10 ^a^	4.01 ± 0.12 ^bc^
BV12	24.17 ± 0.79 ^i^	4.54 ± 0.19 ^c^	4.31 ± 0.11 ^a^	17.00 ± 3.57 ^j^	5.73 ± 0.29 ^ab^	4.13 ± 0.09 ^ab^
BV13	16.00 ± 1.22 ^j^	3.46 ± 0.05 ^c^	4.31 ± 0.07 ^a^	12.97 ± 3.15 ^j^	3.75 ± 0.72 ^c^	4.37 ± 0.17 ^a^

Legend: AS—aseptic system; NAS—non-aseptic system; GC—germination capacity; NL—number of leaves; HL—hypocotyl length; BV1—100% perlite; BV2—100% agar; BV3—100% banana peel; BV4—75% perlite:25% agar; BV5—50% perlite:50% agar; BV6—25% perlite:75% agar; BV7—75% perlite:25% banana peel; BV8—50% perlite:50% banana peel; BV9—25% perlite:75% banana peel; BV10—100% peat; BV11—75% perlite:25% peat; BV12—50% perlite:50% peat, BV13—25% perlite:75% peat; n.a.—not available. All data are expressed as the mean ± standard deviation, *n* = 3. Different letters attributed to the same determination indicate a significant difference between the experimental variants (*p* > 0.05).

**Table 2:** The influence of cultivation substrate on biochemical compound accumulation.

Cultivation System		DM%	Chl *a* (mg/g)	Chl *b* (mg/g)	Total Chl (*a* + *b*) (mg/g)	Chl *a*/*b*	TPC (mg GAE/g)	TAC (mg TE/g)	TFC (mg RE/g)
BV1	AS	6.11 ± 0.05 ^ef^	10.20 ± 0.22 ^d^	8.29 ± 0.12 ^ef^	18.49 ± 0.23 ^ef^	1.23 ± 0.03 ^b^	1.34 ± 0.04 ^bcd^	17.93 ± 2.18 ^def^	0.0034 ± 0.0002 ^c^
	NAS	4.70 ± 0.28 ^a^	9.81 ± 0.31 ^d^	7.41 ± 0.32 ^cde^	17.21 ± 0.68 ^de^	1.32 ± 0.03 ^def^	1.34 ± 0.18 ^bcd^	15.66 ± 6.31 ^defgh^	0.0033 ± 0.0001 ^cf^
BV2	AS	6.29 ± 0.05 ^fg^	9.14 ± 0.41 ^c^	7.11 ± 0.27 ^bcd^	16.26 ± 0.69 ^cd^	1.28 ± 0.01 ^bcde^	1.45 ± 0.08 ^bcd^	18.97 ± 1.41 ^defg^	0.0034 ± 0.0001 ^cd^
	NAS	4.87 ± 0.03 ^ab^	9.31 ± 0.45 ^bc^	7.01 ± 0.19 ^bc^	16.32 ± 0.64 ^cd^	1.33 ± 0.04 ^ef^	1.46 ± 0.06 ^cd^	21.77 ± 1.85 ^efg^	0.0042 ± 0.0001 ^g^
BV4	AS	5.63 ± 0.03 ^de^	10.57 ± 0.21 ^d^	8.45 ± 0.07 ^ef^	19.02 ± 0.42 ^f^	1.25 ± 0.01 ^bc^	1.67 ± 0.16 ^d^	14.98 ± 2.78 ^bcd^	0.0024 ± 0.0001 ^b^
	NAS	6.43 ± 0.17 ^fg^	9.07 ± 0.11 ^bc^	6.77 ± 0.14 ^abc^	15.83 ± 0.21 ^bcd^	1.34 ± 0.01 ^ef^	1.11 ± 0.14 ^b^	20.99 ± 1.98 ^efg^	0.0042 ± 0.0001 ^g^
BV5	AS	5.84 ± 0.21 ^ef^	7.91 ± 0.12 ^a^	6.23 ± 0.00 ^ab^	14.15 ± 0.25 ^ab^	1.27 ± 0.01 ^bcd^	1.49 ± 0.21 ^cd^	17.18 ± 1.59 ^de^	0.0033 ± 0.0002 ^c^
	NAS	5.19 ± 0.27 ^abcd^	7.98 ± 0.19 ^a^	6.00 ± 0.18 ^a^	13.98 ± 0.33 ^a^	1.33 ± 0.01 ^ef^	1.19 ± 0.06 ^bc^	22.98 ± 1.38 ^gh^	0.0038 ± 0.0001 ^ef^
BV6	AS	6.89 ± 0.29 ^g^	12.22 ± 0.55 ^e^	10.48 ± 0.81 ^g^	22.70 ± 1.17 ^g^	1.17 ± 0.02 ^a^	1.56 ± 0.18 ^d^	15.69 ± 0.66 ^cd^	0.0038 ± 0.0001 ^de^
	NAS	4.99 ± 0.19 ^abc^	9.06 ± 0.18 ^bc^	6.72 ± 0.13 ^abc^	15.78 ± 0.28 ^bcd^	1.35 ± 0.01 ^f^	1.22 ± 0.04 ^bc^	26.00 ± 1.79 ^i^	0.0044 ± 0.0001 ^g^
BV10	NAS	5.50 ± 0.45 ^bcde^	10.48 ± 0.03 ^d^	8.02 ± 0.20 ^def^	18.51 ± 0.15 ^ef^	1.31 ± 0.02 ^cdef^	0.73 ± 0.05 ^a^	22.23 ± 1.16 ^fgh^	0.0033 ± 0.0001 ^c^
BV11	NAS	6.04 ± 0.09 ^ef^	7.79 ± 0.23 ^a^	5.94 ± 0.29 ^a^	13.74 ± 0.52 ^a^	1.31 ± 0.02 ^def^	0.58 ± 0.04 ^a^	10.74 ± 0.92 ^ab^	0.0023 ± 0.0002 ^b^
BV12	NAS	5.55 ± 0.21 ^cde^	8.61 ± 0.15 ^ab^	6.35 ± 0.19 ^ab^	14.96 ± 0.31 ^abc^	1.36 ± 0.01 ^f^	0.50 ± 0.01 ^a^	9.95 ± 0.23 ^ab^	0.0021 ± 0.0001 ^ab^
BV13	NAS	5.51 ± 0.18 ^cde^	8.55 ± 0.35 ^ab^	6.58 ± 0.23 ^abc^	15.13 ± 0.98 ^abc^	1.30 ± 0.09 ^ef^	0.41 ± 0.06 ^a^	11.92 ± 1.30 ^ac^	0.0018 ± 0.0001 ^a^

Legend: DM (%)—dry matter; Chl *a*—chlorophyll *a*; Chl *b*—chlorophyll *b*; TPC—total phenolic content; GAE—gallic acid equivalents; TAC—total antioxidant capacity; TE—Trolox equivalents; TFC—total flavonoid content; RE—rutin equivalents; AS—aseptic system; NAS—non-aseptic system; BV1—100% perlite; BV2—100% agar; BV4—75% perlite:25% agar; BV5—50% perlite:50% agar; BV6—25% perlite:75% agar; BV10—100% peat; BV11—75% perlite:25% peat; BV12—50% perlite:50% peat, BV13—25% perlite:75% peat. All values, besides DM, are reported to FW. All data are expressed as the mean ± standard deviation, n = 3. Values with the same letters in a column do not differ significantly (*p* > 0.05).

**Table 3:** The impact of substrates under both AS and NAS conditions (BV1-BV13) on mineral accumulation in basil microplants (reported to FW).

Cultivation System		Na (mg/Kg)	Mg (mg/Kg)	P (mg/Kg)	K (mg/Kg)	Ca (mg/Kg)	Mn (mg/Kg)	Cu (mg/Kg)	Zn (mg/Kg)
BV1	AS	1018.51 ± 23.64 ^h^	261.30 ± 10.21 ^ab^	716.56 ± 44.42 ^cde^	995.65 ± 66.41 ^a^	141.98 ± 0.43 ^a^	2.42 ± 0.09 ^a^	1.79 ± 0.06 ^ab^	7.55 ± 0.19 ^a^
	NAS	502.41 ± 28.09 ^f^	325.06 ± 8.95 ^bc^	606.24 ± 9.20 ^abc^	1374.32 ± 88.90 ^b^	253.40 ± 25.22 ^a^	2.71 ± 0.05 ^a^	1.53 ± 0.03 ^ab^	12.54 ± 0.34 ^c^
BV2	AS	716.83 ± 61.64 ^g^	272.10 ± 30.37 ^ab^	689.79 ± 38.06 ^cd^	1143.21 ± 26.06 ^ab^	160.03 ± 21.59 ^a^	2.16 ± 0.09 ^a^	1.73 ± 0.06 ^ab^	7.43 ± 0.19 ^a^
	NAS	360.71 ± 28.41 ^d^	311.31 ± 26.76 ^b^	655.71 ± 40.28 ^bc^	1426.63 ± 60.17 ^bc^	287.96 ± 7.88 ^a^	3.05 ± 0.23 ^a^	1.78 ± 0.04 ^ab^	12.57 ± 0.61 ^bc^
BV4	AS	1210.64 ± 63.33 ^ij^	288.94 ± 10.50 ^ab^	795.22 ± 22.18 ^def^	1351.45 ± 116.79 ^b^	152.94 ± 4.16 ^a^	2.88 ± 0.28 ^a^	2.23 ± 0.06 ^abc^	8.83 ± 0.52 ^a^
	NAS	511.46 ± 30.22 ^f^	404.81 ± 18.95 ^c^	845.24 ± 28.16 ^f^	1752.55 ± 166.64 ^c^	293.09 ± 12.95 ^a^	3.86 ± 0.32 ^a^	2.29 ± 0.08 ^bc^	16.01 ± 0.18 ^d^
BV5	AS	1296.51 ± 24.67 ^j^	219.30 ± 8.28 ^a^	695.36 ± 17.14 ^cde^	1329.89 ± 75.25 ^ab^	131.85 ± 3.02 ^a^	3.10 ± 0.05 ^a^	1.64 ± 0.10 ^ab^	7.06 ± 0.51 ^a^
	NAS	372.48 ± 30.81 ^d^	269.67 ± 10.48 ^ab^	564.44 ± 24.39 ^ab^	1308.27 ± 58.37 ^ab^	283.62 ± 29.79 ^a^	2.99 ± 0.26 ^a^	1.50 ± 0.12 ^a^	11.30 ± 1.03 ^b^
BV6	AS	1149.00 ± 36.99 ^i^	287.95 ± 22.04 ^ab^	810.98 ± 30.80 ^ef^	1467.40 ± 75.47 ^bc^	228.54 ± 54.86 ^a^	2.67 ± 0.21 ^a^	2.24 ± 0.12 ^abc^	8.53 ± 0.39 ^a^
	NAS	315.25 ± 25.23 ^cd^	257.73 ± 38.71 ^ab^	568.93 ± 48.20 ^ab^	1310.22 ± 105.08 ^ab^	261.00 ± 52.18 ^a^	2.64 ± 0.17 ^a^	1.57 ± 0.09 ^ab^	12.03 ± 0.79 ^bc^
BV10	NAS	45.21 ± 0.50 ^a^	250.94 ± 16.68 ^ab^	537.46 ± 32.21 ^a^	2475.72 ± 90.79 ^d^	711.13 ± 43.14 ^b^	38.56 ± 1.79 ^c^	1.83 ± 0.12 ^a^	8.88 ± 0.82 ^a^
BV11	NAS	561.60 ± 16.95 ^f^	606.67 ± 29.64 ^e^	603.59 ± 40.69 ^abc^	2166.33 ± 100.64 ^d^	1872.45 ± 92.64 ^d^	27.94 ± 1.89 ^b^	2.49 ± 0.07 ^c^	˂LOD
BV12	NAS	224.11 ± 13.77 ^bc^	517.08 ± 45.72 ^d^	542.66 ± 58.53 ^a^	2961.41 ± 192.20 ^e^	1486.45 ± 93.77 ^c^	36.30 ± 1.96 ^bc^	2.27 ± 0.25 ^ac^	˂LOD
BV13	NAS	150.00 ± 10.96 ^b^	538.97 ± 57.60 ^de^	618.53 ± 68.74 ^abc^	3816.39 ± 223.86 ^f^	1711.23 ± 314.59 ^cd^	44.42 ± 0.58 ^d^	2.80 ± 0.17 ^c^	˂LOD

Legend: AS—aseptic system; NAS—non-aseptic system; BV1—100% perlite; BV2—100% agar; BV4—75% perlite:25% agar; BV5—50% perlite:50% agar; BV6—25% perlite:75% agar; BV10—100% peat; BV11—75% perlite:25% peat; BV12—50% perlite:50% peat, BV13—25% perlite:75% peat; Na—sodium; Mg—magnesium; P—phosphorous; K—potassium; Ca—calcium; Mn—manganese; Cu—copper; Zn—Zinc; LOD—limit of detection. All data are expressed as the mean ± standard deviation, n = 3. Values with the same letters in a column do not differ significantly (*p* > 0.05).

**Table 4:** Microbial contaminants on the basil microplants.

Variants	AS	NAS
BV1	Uncontaminated	*Phoma* sp.
BV2	Uncontaminated	*Phoma* sp.
BV3	*Acremonium* sp., *Curvularia* sp.	*Phoma* sp.
BV4	Uncontaminated	*Penicillium* sp.
BV5	Uncontaminated	*Penicillium* sp.
BV6	Uncontaminated	Rod bacteria
BV7	*Penicillium* sp.	Rod bacteria and *Phoma* sp.
BV8	Uncontaminated	*Phoma* sp.
BV9	Uncontaminated	*Phoma* sp. and *Aspergillus* sp.
BV10	Uncontaminated	Fungal, bacterial, and microalgal community
BV11	Uncontaminated	*Fusarium* sp.
BV12	Uncontaminated	*Fusarium* sp.
BV13	*Fusarium* sp.	*Fusarium* sp.

**Table 5:** Variations in cultivation substrate for basil microplants.

Variants Cod	Perlite	Agar	Banana Peels	Conventional Substrate (Peat)
BV1	100%	-	-	-
BV2	-	100%	-	-
BV3	-	-	100%	-
BV4	75%	25%	-	-
BV5	50%	50%	-	-
BV6	25%	75%	-	-
BV7	75%	-	25%	-
BV8	50%	-	50%	-
BV9	25%	-	75%	-
BV10	-	-	-	100%
BV11	75%	-	-	25%
BV12	50%	-	-	50%
BV13	25%	-	-	75%

## Data Availability

The original contributions presented in the study are included in the article, further inquiries can be directed to the corresponding author.
